# Contribution to Charophyceae knowledge in Slovakia – re-discovery of *Nitella gracilis*

**DOI:** 10.3897/BDJ.14.e197476

**Published:** 2026-08-10

**Authors:** František Bednár, Martin Dobrota, Roman Romanov, Richard Hrivnák

**Affiliations:** 1 Lániky 271/26, Švošov, Slovakia Lániky 271/26 Švošov Slovakia; 2 Slobody 313/23, Turčianske Teplice, Slovakia Slobody 313/23 Turčianske Teplice Slovakia; 3 Dobra Voda, Bar, Montenegro Dobra Voda Bar Montenegro; 4 Institute of Botany, Plant Science and Biodiversity Center, Slovak Academy of Sciences, Bratislava, Slovakia Institute of Botany, Plant Science and Biodiversity Center, Slovak Academy of Sciences Bratislava Slovakia

**Keywords:** ecology, macroscopic algae, *Nitelletum gracilis*, rare species, stoneworts

## Abstract

**Background:**

*Nitella gracilis* (Charophyceae) is a rare charophyte, previously considered regionally extinct in Slovakia. During a systematic survey of charophytes conducted between 2021 and 2025, new localities of this species were discovered. These findings represent the first confirmed records of the species in Slovakia in more than 45 years. The results highlight the importance of small temporary waterbodies such as forest road puddles for the conservation of charophytes.

**New information:**

We found *Nitella gracilis* in two localities. The first locality was found in a forest pool in the Krupinská planina Mountains, while the second consisted of 23 microsites, located in road puddles near the village of Sklené in the Turčianska kotlina Basin. The water in pools supporting *N. gracilis* ranged from slightly acidic to slightly alkaline (pH 5.7–7.5) and exhibited relatively low electrical conductivity (21–274 μS/cm). At the second locality, phytosociological relevés were recorded around stands dominated by *N. gracilis* and classed as the association *Nitelletum gracilis*. Based on the limited number of confirmed sites, we propose changing its national Red List status from Regionally Extinct (RE) to Endangered (EN (B1, B2a, c)) (IUCN 2012a, IUCN 2012b, IUCN 2024).

## Introduction

Charophytes, a group of macroscopic algae (stoneworts), are primarily freshwater organisms widely distributed across Europe. Although current knowledge about stoneworts was recently summarised in the comprehensive book *Charophytes in Europe* (Schubert et al. 2024), research on this group remains ongoing and additional data are needed.

The genus *Nitella* C. Agardh 1824 (division Charophyta, class Charophyceae, order Charales) represents a natural group of macroscopic green algae distinguished from other genera of Charales by ecorticate and mostly non-encrusted thalli. They have no stipulodes. The branchlets are segmented, furcate or simple, composed of more or less equal-sized unicellular segments. The terminal segments of branchlets, referred to as dactyls, can be one-celled or two- or more-celled. *Nitella* species can be monoecious or dioecious. The fruiting branchlets are often arranged in large loose or densely packed whorls. Antheridia are located at the apex of the segments in the branchlet forks, while oogonia are lateral. In monoecious species, oogonia are located under the antheridia and oospores are elliptical in cross section. The crown of the oogonia consists of 10 cells arranged in two tiers of five in each (Casanova 2024).

Thirteen species of *Nitella* are known from Europe (Schubert et al. 2024), slightly more than half of which have been recorded in Slovakia (Hindáková et al. 2022). All species of the genus *Nitella* are poorly known and rare in Slovakia. During the latest critical review of charophytes, 252 herbarium specimens of Charophytes (Characeae) were examined. Seven species of *Nitella* were identified, represented by only 20 records: *Nitella mucronata* (A. Braun) Miq., *Nitella gracilis* (Smith) C. Agardh (see Table 1), *Nitella flexilis* (L.) C. Agardh, *Nitella syncarpa* (Thuill.) Chev., *Nitella capillaris* (Krock.) J. Groves & Bull.-Webst., *Nitella opaca* (C. Agardh ex Bruzelius) C. Agardh and *Nitella confervacea* (Bréb.) A. Braun ex Leonh. Of the total number of records, only eight date from the 21^st^ century, with the most recent being from 2002. *Nitella mucronata* and *Nitella syncarpa* are classed as Endangered (EN) in Slovakia, while the remaining five *Nitella* species are classed Regionally Extinct (RE) (Hindáková et al. 2022).

One of the *Nitella* species, classed as Regionally Extinct (RE) in Slovakia, is *Nitella gracilis*, a cosmopolitan species that is widely distributed across Europe, although it is not common. Its distribution is more frequent in the western and central parts of the continent, while the number of localities decreases towards the southern, northern and eastern regions (Gąbka & Urbaniak in Schubert et al. (2024). In the countries surrounding Slovakia, the conservation status of this species is as follows: Critically Endangered in Poland (Urbaniak and Gąbka 2014), Vulnerable in Ukraine, where it has been recorded from eight localities, five of which have been confirmed since 2000 (Borysova 2024), Endangered in Austria with nine known localities, eight of them recorded in the recent period (Raabe 2024, Hohla et al. 2025) and Highly Endangered in the Czech Republic, where 18 localities are known, only four of which have been confirmed in recent years (Caisová and Gąbka 2009). Although the species is known from south-western and western Transdanubia, Hungary (Schubert et al. 2024), *Nitella gracilis* is not included in the Hungarian Red List (Németh 2005). Patterns of distribution, historical occurrence and threats differ amongst the countries studied. For example, there were eight occurrences in Central Burgenland in Austria in 2022–2023. Before that, *Nitella gracilis* was last recorded in Austria in 1970 in the Wienerwald (Vienna Woods) in Lower Austria (M. Hohla, pers. comm.). The recent discoveries in Burgenland suggest the species may still be present in other parts of Austria, but has likely been overlooked, especially considering its occurrence in small or very small, often transient waterbodies (Raabe 2024).

*Nitella gracilis* is known exclusively from freshwater habitats, including lakes, ponds, pools, ditches and clay pits, occurring in waters ranging from shallow to deep and from acidic to slightly alkaline conditions. *Nitella gracilis* is a pioneer species of small to very small waterbodies and species competitive interactions rather than nutrient availability appear to influence its distribution (Hohla et al. 2025). The community dominated by *Nitella gracilis* was described by Corillion as the association *Nitelletum gracilis* Corillion 1957, but it is very rarely recorded and documented in Europe (cf. Schubert et al. (2024).

In recent years, we have obtained new data on the distribution, ecology and phytosociology of *Nitella gracilis*. Therefore, the aims of this paper are: (i) to describe the distribution of the re-discovered charophyte *Nitella gracilis* and (ii) to document, for the first time in Slovakia, the community dominated by this species, *Nitelletum gracilis*.

## Materials and methods

*Nitella gracilis* was discovered during a systematic search for charophytes in Slovakia during the seasons 2021–2025. Around 200 prospective aquatic habitats were visited and searched visually and/or with grapnel, both from the shore and/or from a boat. Most of the habitats with historical records were also visited. The findings were photographically documented in the field, samples were collected and later photographed in a home laboratory by Canon EOS R7 and Canon EF 100/2.8 USM lens. The material was examined microscopically by Zeiss Stemi 305 and Olympus BX51 microscopes. Microscope slides were prepared from living specimens mounted in tap water without staining. Microphotographs were taken using the Canon EOS R7 camera and dimensions were determined from the images via a calibrated slide (Zeiss Calibration Slide Universal CG=0, Item no.: 474029-9011-000).

Specimens were identified using standard identification keys by Krause (1997), Caisová and Gąbka (2009), Urbaniak and Gąbka (2014), Schubert et al. (2024).

Several individuals of *Nitella gracilis* were selected for herbarium specimens. Herbarium sheets were prepared in accordance with the guidelines of the Herbarium Handbook (Royal Botanic Gardens Kew 2019). Specimens are deposited in the first author’s private herbarium (HBE) and in the Plant Science and Biodiversity Centre, Slovak Academy of Sciences herbarium (SAV).

The area of each site was measured on satellite maps in Google Earth (https://earth.google.com/). Water depth was recorded at every location using several randomly selected measurements. Water temperature, pH and conductivity were measured *in situ* near the water surface (water depth 5–10 cm) using a portable Thermo Scientific™ Elite PCTS meter, calibrated to Fischer NIST buffer standard solutions.

All observations are available at iNaturalist https://www.inaturalist.org/observations?d2=2026-02-22&taxon_id=486281&user_id=fero&verifiable=any (accessed 2.2.2026).

Distribution maps were prepared in Google My Maps (https://www.google.com/).

The vegetation dominated by *Nitella gracilis* was studied using the traditional Zürrich-Montpellier's approach (Westhoff and van der Maarel 1973), with a modified Braun-Blanquet scale (Barkman et al. 1964).

The names of charophytes and vascular plants follow AlgaeBase (www.algaebase.org) and Plants of the World Online (https://powo.science.kew.org/), respectively. The names of syntaxa above the rank of association and nomenclature of vascular plants follow Mucina et al. (2016) and Letz et al. (2024), respectively.

## Taxon treatments

### Nitella gracilis

C. Agardh, 1824

4380611C-C330-59DC-957E-94A8F5BD2B80

https://www.algaebase.org/search/species/detail/?species_id=35596

#### Materials

- **Type status:** Other material. **Occurrence:** occurrenceID: A0083228-0A1F-5DB3-98DC-CC9A0E17A6D0; **Taxon:** taxonID: https://www.gbif.org/species/2637830; scientificName: *Nitella gracilis* (Sm.) C.Agardh; **Location:** country: Slovakia; locality: Krupinská planina Mts. near the road to Čabraď Castle; verbatimElevation: 406; verbatimLatitude: 48 13 55.9 N; verbatimLongitude: 19 06 00.2 E; **Identification:** identifiedBy: František Bednár; **Event:** year: 2024; month: June; day: 22
- **Type status:** Other material. **Occurrence:** occurrenceID: 8F0A4577-7D75-51ED-879B-E3178567DB38; **Taxon:** taxonID: https://www.gbif.org/species/2637830; scientificName: *Nitella gracilis* (Sm.) C.Agardh; **Location:** country: Slovakia; locality: Turčianska kotlina basin, north of the Sklené village in Turčianska kotlina basin, "Rovná hora”; verbatimElevation: 624; verbatimLatitude: 48 48 50.9N; verbatimLongitude: 18 50 43.8E; **Identification:** identifiedBy: František Bednár and Martin Dobrota; **Event:** year: 2025; month: August; day: 7

#### Description

**Figure 1.**
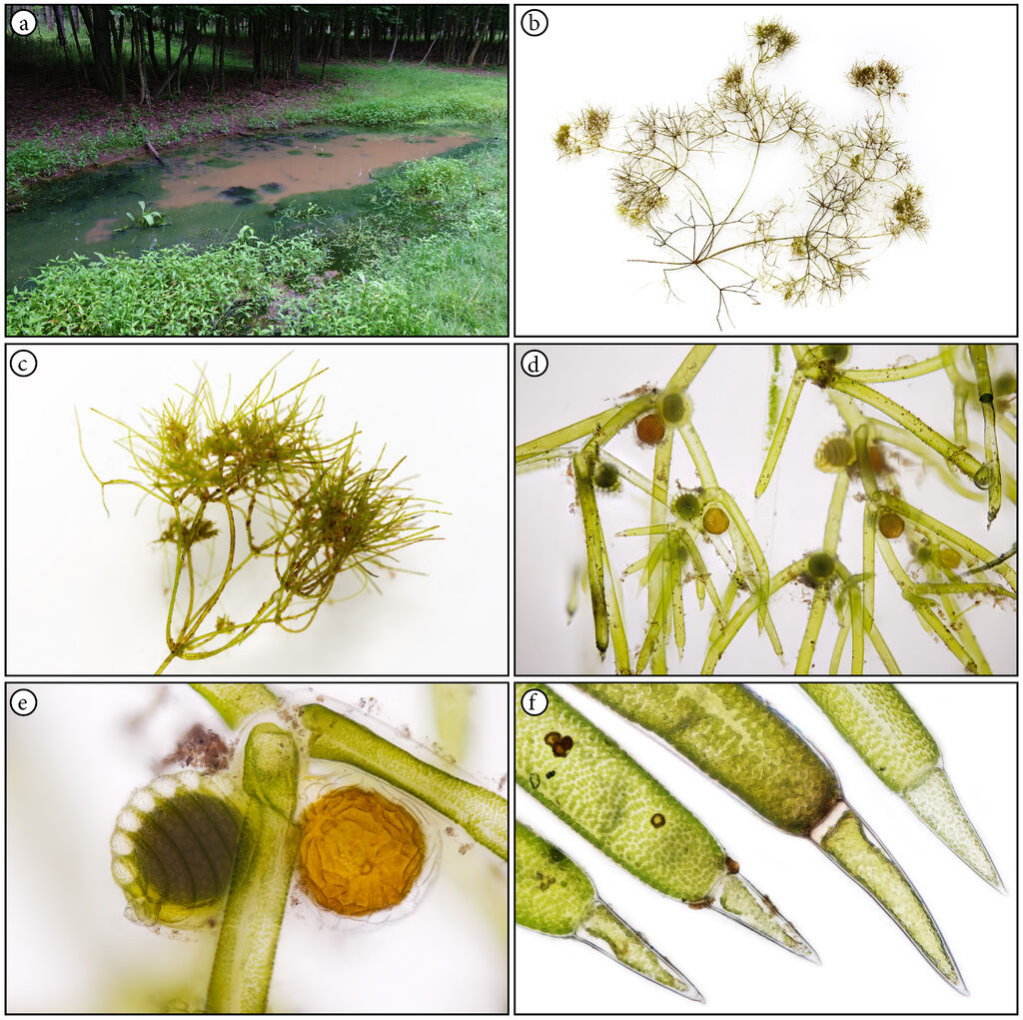
*Nitella gracilis*, 22 June 2024, Čabraďský vrbovok, Krupinská planina, Slovakia. **a** locality (Nitellas are dark green in the middle); **b** algae habitus; **c, d** details of a whorl; **e** oogonium and antheridium; **f** detail of terminal cells; photos by F. Bednár.

**Figure 2.**
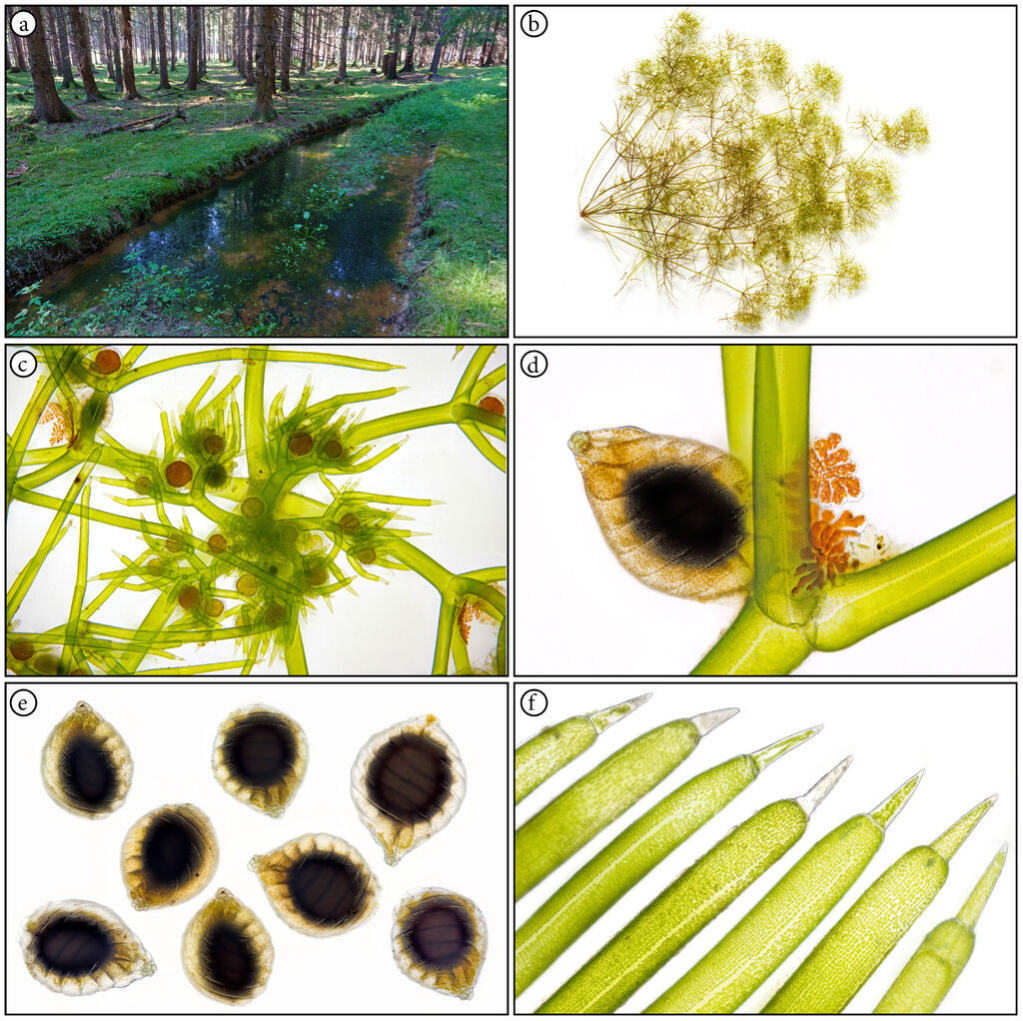
*Nitella gracilis*, 7 August 2025, Rovná hora, Sklené, Turčianska kotlina, Slovakia. **a** locality (Nitellas are dark green); **b** algae habitus; **c** details of a whorl; **d** details of gametangia; **e** oospores; **f** detail of terminal cells; photos by F. Bednár.

The found *Nitella gracilis* plants were monoecious, hosting both antheridia and oogonia and were growing in a group of several individuals together, light yellow green to green in colour, slender and with delicate branches, forming whorls, resembling small *Nitella mucronata* in their habitus. The terminal cells of dactyls were about 2/3 as broad as the subapical cell. Fertile whorls formed heads. Oospores had punctate (granulate) oospore wall ornamentation (see Figs 1, 2). These features are diagnostic against the most similar Slovak congener, *Nitella mucronata*, which differs in having a short, conical, mucronate end-cell about 1/3 as broad as the subapical cell (versus the acute end-cell, about 2/3 as broad as the subapical cell, found in our specimens), a generally more robust habit and reticulate rather than punctate oospore wall ornamentation (Schubert et al. 2024). Very small, delicate individuals could potentially be confused with *Nitella confervacea*, but the upper whorls of our specimens were not contracted and the branchlet end segments were mostly 3-celled, whereas in *N. confervacea* the end segments are always 2-celled (Schubert et al. 2024).

#### Distribution

**Figure 3.**
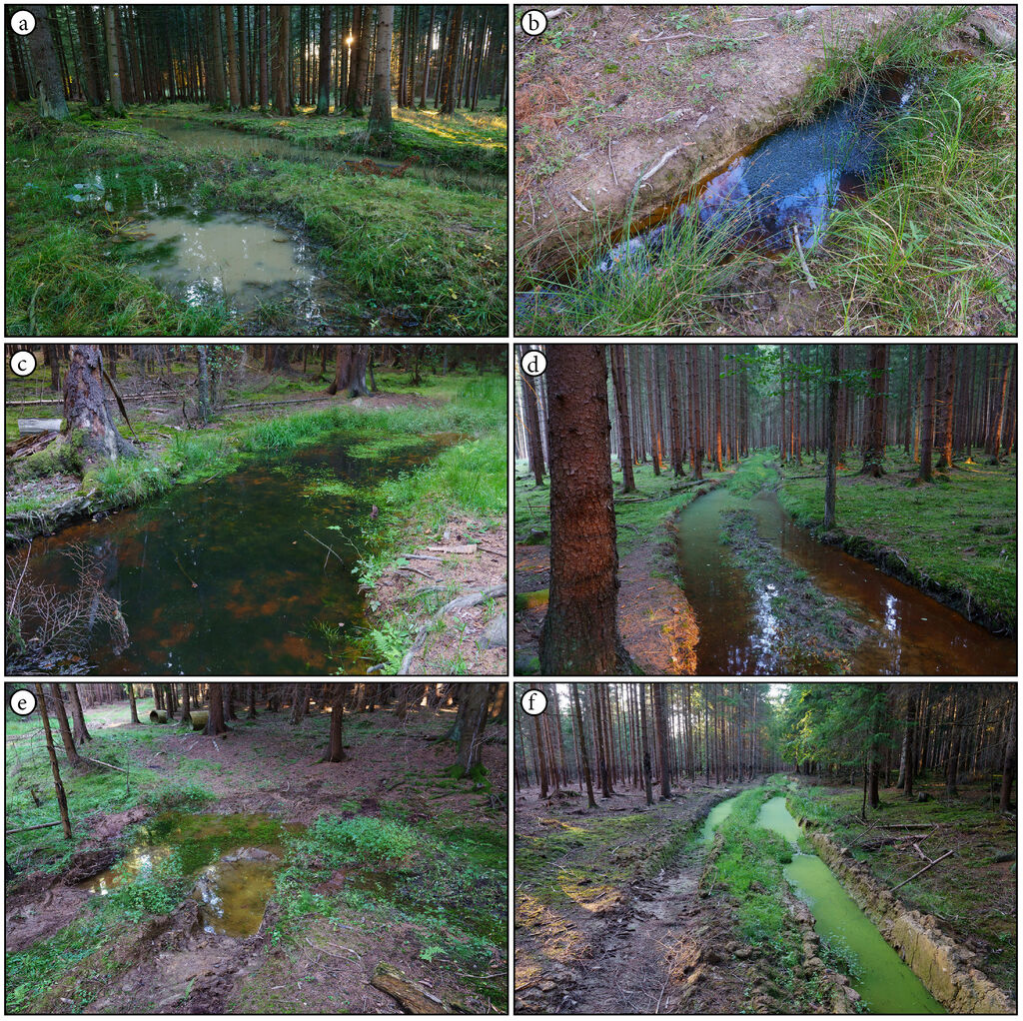
**a-f** various road track puddle locations of *Nitella gracilis* within Rovná hora. The depressions and linear features were formed by repeated passage of forestry machinery, which created compacted soil ruts and shallow channels. Due to the predominance of clay-rich soils, water is retained in these depressions for extended periods, resulting in a mosaic of small, semi-permanent to permanent waterbodies. These include shallow pools, interconnected ruts and linear drainage tracks with stagnant or very slow-flowing water. The sites are strongly shaded by the surrounding spruce forest and are characterised by soft, organic- and clay-rich substrates, with limited surface runoff and high water retention. This creates suitable conditions for the persistence of small aquatic habitats within an otherwise terrestrial forest environment; photos by F. Bednár.

The first new location of *Nitella gracilis* was found in Krupinská planina Mts. near the road to Čabraď Castle (for more detail see Materials and Methods, a); Fig. 1). The second location of *Nitella gracilis* was found in “Rovná hora”, north of the Sklené village in Turčianska kotlina Basin (see Materials and Methods, b); Fig. 2 and Fig. 3).

#### Ecology

In the Krupinská planina Mts., *Nitella gracilis* grew in a water-filled pool in a clearing within mixed deciduous forest, mainly oak-hornbeam and beech stands, in a semi-shaded area at the edge of young *Fagus sylvatica* trees and the road. The pool was 10–20 cm deep, approximately 20 m^2^ area, water was muddy and of a yellow-white appearance with temperature 26.3°C, pH 7.41 and conductivity 135 μS/cm.

**Figure 4.**
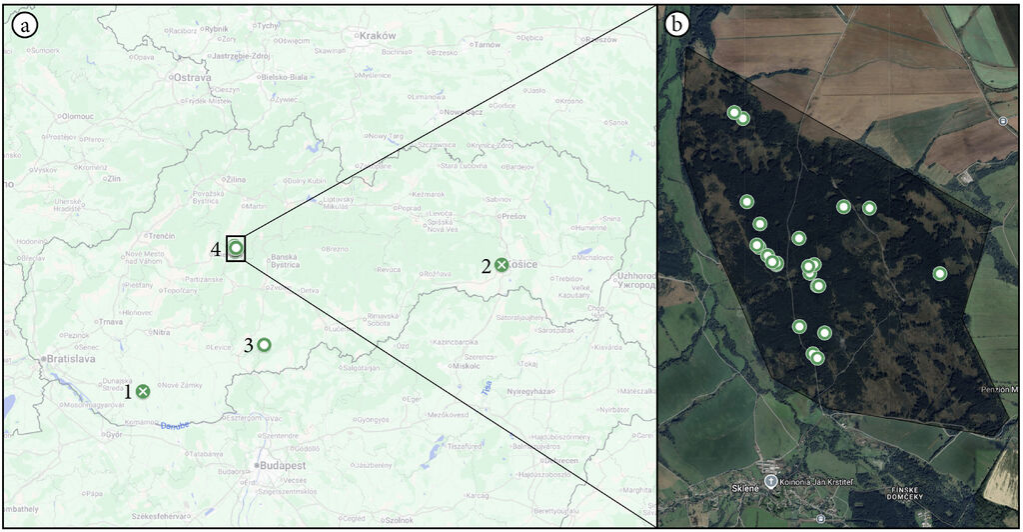
Distribution of *Nitella gracilis* in Slovakia (a), 1) 1953 Komárno, 2) 1959 Košice, both historical and unconfirmed records, 3) 2024 Krupinská planina, 4) 2025 Rovná hora and distribution of microsites in the forest of Rovná hora (b). Base map and imagery: © Google Maps.

In Rovná hora, *Nitella gracilis* grew in a pool along a track 2 m × 8 m, at a depth of 10–30 cm, covering approximately 70% of the surface area. From 7 August 2025 to 7 November 2025, a systematic search of the wood found a total of 23 microsites (Fig. 4), all in road puddles (Fig. 3), within 1 km radius, 30–500 m apart from each other, with a total water area of 349 m^2^, coverage of 79 m^2^ by *Nitella gracilis* (1–90% coverage of individual sites). The average pH from all sites was 6.6 (from slightly acid to neutral, 5.7–7.5) and average conductivity 78.5 μS/cm (21.2–274).

#### Biology

In the Krupinská planina Mts. four clumps of *Nitella gracilis* were found growing in the central part of the puddle, together with the conjugate alga *Spirogyra* sp., *Lemna minor* and *Alisma plantago-aquatica* as well as several hygrophilous species (e.g. *Glyceria notata*, *Juncus effusus*, *Lysimachia nummularia*, *Persicaria hydropiper*, *Ranunculus repens* and *Veronica beccabunga*).

In Rovná hora, the species most frequently recorded accompanying *Nitella gracili*s were *Callitriche cophocarpa* and several hygrophilous species (e.g. *Alisma plantago-aquatica*, *Glyceria fluitans*, *Ranunculus flammula* and *Ranunculus repens*).

Locally, at the second locality in Rovná hora, stands dominated by *Nitella gracilis*, with more frequent occurrence of other hydrophyte and hygrophilous species, were recorded (Table 2, see also Fig. 3). These stands were classified as the association *Nitelletum gracilis*.

## Discussion

The discovery of two new records of *Nitella gracilis* after a gap of 45 years, bringing the total number of records for the country to five, significantly contributes to the knowledge of the taxon and genus in Slovakia. Unfortunately, we were not able to verify historical records as the locations were not sufficiently localised. As historical records could not be verified and only two active localities have been found in Slovakia, we suggest changing the status of *Nitella gracilis* on the Red List of Slovak Charophytes from RE to EN (B1, B2a, c; cf. IUCN (2012a), IUCN (2012b), IUCN (2024)). Nevertheless, recent findings of *Nitella gracilis* from Slovakia suggest that the species might be more common than previously assumed (cf. Raabe 2024, Hohla et al. 2025). It is very likely that more systematic surveys in suitable habitats might reveal additional sites with the occurrence of *Nitella gracilis*.

All records of *Nitella gracilis* in Slovakia were from small waterbodies, particularly pools on tracks. The importance of such habitats for charophytes is well known, yet it is still often underestimated. Our findings support those from Austria and once again demonstrate the necessity of searching for charophytes even in seemingly “unattractive” sites. Some species occur preferentially or even exclusively, in such habitats (Müller and Raabe 2017, Raabe 2024). Most recent Austrian observations are from mud wallows along forest tracks. Older Austrian records from wheel ruts could not be confirmed due to extensive upgrading and surfacing of forest roads (Raabe 2024). In contrast, most Slovak localities are situated in wheel ruts on forest roads. This suggests that small-scale, periodically disturbed microhabitats created by forestry activities may provide suitable conditions for this weakly competitive pioneer species. At the second locality in Rovná hora, where 23 microsites were recorded, ongoing forest management appears to maintain the dynamic conditions necessary for the persistence of the species. These observations indicate that moderate, recurrent disturbance may play an important role in the conservation of *Nitella gracilis*, whereas both habitat stabilisation and eutrophication could negatively affect its long-term survival. The original source habitats of *Nitella gracilis* and similar pioneer charophytes were most likely marsh edges, swamp forest margins and stream backwaters, from which the species may have subsequently colonised anthropogenic substitute habitats, such as forest road ruts and puddles. Small forest pools of this kind are increasingly recognised as important refuges for other species of conservation concern, including the Natura 2000 species *Eleocharis carniolica* and *Bombina bombina* (Haraszthy 2024), underlining the broader conservation value of this habitat type beyond charophytes alone.

Several factors may threaten these habitats and warrant attention in future conservation planning: forest road construction and resurfacing (cf. Raabe (2024)), premature drying following clear-cutting and climatic trends towards longer dry periods. Notably, the same wildlife activity that may aid dispersal (see below) can also threaten existing populations, as animals wallowing in these pools during dry spells can physically destroy the vegetation and, in extreme cases, eliminate a population outright. The persistence of *Nitella gracilis*, therefore, appears to depend on a balance between disturbance and stability: moderate, periodic disturbance from forestry activity favours this weakly competitive species, but disturbance that is too frequent or severe, such as repeated wallowing during drought, is likely detrimental. Shaded, forest-interior pools with soft, clay-rich sediment also tend to retain water and undergo slower succession than open-area puddles, which dry out more quickly; such forest pools may, therefore, represent the only remaining aquatic habitat available to the species during dry periods, allowing populations to persist for several years even in the absence of renewed disturbance.

Based on our field observations, the mechanism by which *Nitella gracilis* colonised these forest-associated microhabitats remains speculative; however, several pathways are plausible. Oospores of charophytes are commonly dispersed by waterfowl and other animals through gut passage or attachment to feathers, fur or feet and the frequent use of forest-road wheel ruts and game watering sites by wildlife (e.g. deer, wild boar, birds) likely provides repeated opportunities for such transport between water bodies (see also Charalambidou and Santamaría (2005), Raabe (2024) and Schubert et al. (2024), although, as noted above, the same wallowing activity can also be locally destructive to existing stands. In addition, forestry machinery operating along these tracks may contribute to local dispersal by transporting mud and water containing oospores or vegetative fragments between sites. However, it is also possible that the species has persisted undetected at these or similar localities for a considerably longer period, given the long-term viability of charophyte oospores in sediment and the limited survey effort historically directed at small, ephemeral habitats, such as roadside puddles and wheel ruts. Under this scenario, the apparent re-discovery would reflect increased survey attention rather than recent colonisation, consistent with our broader argument that the species may be more widespread in Slovakia than current records suggest.

Habitats with *Nitella gracilis* in Slovakia differ from those reported from other European locations, where lakes, ponds, pools, ditches and clay pits are most frequently recorded and sand pits in old riverbeds are recorded more rarely (Schubert et al. 2024). In contrast, the water characteristics recorded in Slovakia, such as pH and conductivity, fall within the range reported from other European countries. We evaluated the habitats as mesotrophic to meso-eutrophic. Most European localities can be classified as oligo-mesotrophic or mesotrophic; however, some are meso-eutrophic to eutrophic (Caisová and Gąbka 2009, Urbaniak and Gąbka 2014, Borysova 2024). The water quality recorded at the newly-discovered Slovak localities ranged from slightly acidic to slightly alkaline, which is narrower than the range generally reported from other European countries (Schubert et al. 2024), but is similar to that reported, for example, from Ukraine (Borysova 2024).

The geological substrate of the two newly-discovered localities differed. The first locality is characterised by andesite, with minor occurrences of dacite, agglomerates/breccias and tuffites, whereas the second is composed of marine molasse deposits, lithologically dominated by claystone with minor siltstone, sandstone and tuffites (Asch 2003). Despite these geological differences, both localities exhibited similar habitat and, to some extent, ecological characteristics. Soil with a high clay content, together with shading by trees, provided suitable conditions for the long-term persistence of pools along tracks.

The species composition of *Nitelletum gracilis* in Slovakia, based on data from the Rovná hora location, fully reflects the habitat characteristics of its localities. Small, narrow and permanently waterlogged tracks shaded by trees allow the co-existence of hydrophytes (*Nitella gracilis* and *Callitriche cophocarpa*) and hygrophytes (most frequently *Alisma plantago-aquatica* and *Glyceria fluitans*). The species composition of this association is similarly heterogeneous in Central and South-Eastern Europe; however, true aquatic plants are always present there and usually exhibit higher species richness. For example, in Poland, the association occurs mainly in lakes and ponds, from shallow to relatively deep water (up to 3 m). Consequently, true aquatic plants of the class Potamogetonetea are consistently present in these stands, while hygrophytes are absent (Tomaszewicz 1979, Urbaniak and Gąbka 2014). In contrast, in Romania, *Alisma plantago-aquatica* is frequently present in the stands of this association in addition to aquatic species (Coldea 1997). In addition, monodominant stands are often recorded (e.g. Iakushenko and Borysova (2012), Urbaniak and Gąbka (2014).

The new findings of *Nitella gracilis* in Slovakia demonstrate that the species is not extinct, but has been re-discovered in the country within specific habitats. Moreover, the locality in Rovná hora with an estimated total coverage of 79 m^2^ represents one of the most significant sites for the species in Central and Eastern Europe. Together with the associated ecological data, these findings help to fill a gap in the knowledge of the species’ distribution and environmental requirements in Central Europe. In addition, the results of our study may stimulate further research on stonewort in Slovakia.

### Conflict of interests

The authors declare that they have no conflicts of interest regarding the publication of this manuscript or the research presented in it.

### Data Availability

All occurrence records of *Nitella gracilis* reported in this study are publicly available through iNaturalist (https://www.inaturalist.org/observations?d2=2026-02-22&taxon_id=486281&user_id=fero&verifiable=any) and are mobilised to GBIF via the iNaturalist Research-grade Observations dataset. Environmental measurements (water depth, temperature, pH, conductivity) and the phytosociological relevés of the association *Nitelletum gracilis* are provided in Tables 1 and 2 of this manuscript and will be archived on the Biodiversity Data Journal/ARPHA platform upon publication. Voucher specimens are listed under Materials and Methods with their respective occurrenceIDs.

## Supplementary Material

XML Treatment for Nitella gracilis

## Figures and Tables

**Table 1.** Historical records of *Nitella gracilis* in Slovakia (all herbarium specimens were revised by M. Gąbka).

	**Location (original description)**	**Collector**	**Source**
25 June 1953	Komárno District (CSR. Slovakia merid., district Komárno)	Hejný, S.	POZ (Herbarium - Adam Mickiewicz University in Poznań, Poland); id. I. Dąmbska and M. Gąbka; Ramienice Czechosłowacji 12/1
1959	Fish pond, Košice (Rybnik k. Košic)	Dąmbska, I.	POZ; id. M. Gąbka; not catalogued
1959	Around Košice (Koło Koszyc)	Dąmbska, I.	POZ id. M. Gąbka; not catalogued

**Table 2.** Relevés of *Nitelletum gracilis* in Slovakia. Localities of relevés: 1. Turčianska kotlina Basin, Sklené Village, north of the village, Rovná hora, road puddles with standing water in secondary spruce forests (hereafter only Sk), 48°48'50.90"N, 18°50'43.80"E, altitude 548 m a.s.l., water depth 1–10 cm, relevé area 15 m², total cover 95%; 2. Sk, 48°48'6.88"N, 18°50'19.22"E, 565 m a.s.l., 15–30 cm, 7 m², total cover 80%; 3. Sk, 48°47'58.11"N, 18°50'27.28"E, 572 m a.s.l., 20–60 cm, 8 m², 95%; 4. Sk, 48°48'31.38"N, 18°50'4.42"E, 559 m a.s.l., 20–30 cm, 5 m², 98%. The author of all relevés is R. Hrivnák; sampled on the 22 September 2025.

**Taxon / Relevé number**	**1**	**2**	**3**	**4**
*Nitella gracilis*	5	4	5	5
*Callitriche cophocarpa*	+	1	+	a
*Glyceria fluitans*	r	1	1	.
*Alisma plantago-aquatica*	1	a	1	.
*Persicaria hydropiper*	+	.	r	.
*Ranunculus flammula*	.	1	+	.
*Ranunculus repens*	.	r	.	r
*Bidens frondosa*	+	.	.	.
*Myosotis palustris* agg.	.	r	.	.
*Scirpus sylvaticus*	.	.	.	r
